# The usefulness of chemical-shift magnetic resonance imaging for the evaluation of osteoid osteoma

**DOI:** 10.1590/0100-3984.2017.0037

**Published:** 2018

**Authors:** Flávia Martins Costa, Clarissa Canella, Filipa Gomes Vieira, Evandro Miguelote Vianna, Walter Meohas, Edson Marchiori

**Affiliations:** 1 MD, PhD, Clínica de Diagnóstico Por Imagem (CDPI), Rio de Janeiro, RJ, Brazil.; 2 MD, PhD, Clínica de Diagnóstico Por Imagem (CDPI), Rio de Janeiro, RJ, and Universidade Federal Fluminense (UFF), Niterói, RJ, Brazil.; 3 MD, Hospital de Braga, Braga, Portugal.; 4 MD, Instituto Nacional de Traumatologia e Ortopedia (INTO), Rio de Janeiro, RJ, Brazil.; 5 MD, PhD, Universidade Federal do Rio de Janeiro (UFRJ), Rio de Janeiro, RJ, Brazil.

**Keywords:** Osteoma, osteoid, Magnetic resonance imaging, Neoplasms, bone tissue

## Abstract

**Objective:**

The purpose of this study was to determine whether chemical-shift magnetic resonance imaging (MRI) could be useful in the diagnosis of osteoid osteoma when clinical and radiological tumor features are inconclusive.

**Materials and Methods:**

This retrospective study included 17 patients who underwent chemical-shift MRI for the evaluation of osteoid osteoma. For all patients, two musculoskeletal radiologists independently recorded signal intensities on in-phase and out-of-phase images in the nidus of the tumor, in abnormal-intensity bone marrow surrounding the lesion, and in normal-appearing bone marrow. For each region, relative signal intensity ratios were calculated by dividing out-of-phase by in-phase values. Relative ratios > 1 were considered indicative of neoplastic lesions. Statistical analysis was carried out to analyze the sample. Inter-observer and intra-observer agreement for each imaging method were assessed using intraclass correlation coefficients according to the Fleiss method and a value > 0.65 was considered to indicate substantial agreement.

**Results:**

The mean relative signal intensity ratios were 1.2 (range, 0.9-1.4) for the nidus and 0.35 (range, 0.11-0.66) for the surrounding tissue; these values differed significantly from the relative signal-intensity ratios for normal-appearing bone marrow (*p* < 0.05).

**Conclusion:**

Chemical-shift MRI is useful for the diagnosis and evaluation of osteoid osteoma.

## INTRODUCTION

Osteoid osteoma is the third most common primary benign skeletal neoplasm; it occurs frequently in young patients and shows a predilection for males^(^^1^^)^. Most patients experience pain that worsens at night and is relieved by the administration of nonsteroidal anti-inflammatory drugs. Osteoid osteoma is an osteolytic defect with sharp margins and a vacularized nidus, which may be surrounded by marginal sclerosis and cortical thickening^(^^2^^-^^7^^)^.

The primary purpose of the diagnostic investigation is detection of the nidus, by modern methods if necessary, to avoid inappropriate treatment^(^^8^^)^. The imaging features of osteoid osteoma are frequently confused with those of Ewing’s sarcoma or chronic osteomyelitis, especially Brodie’s abscess, because of the extensive periosteal reaction, cortical thickening, and sclerosis^(^^9^^)^. In most cases, the nidus can be identified on computed tomography (CT), although CT has been associated with disadvantages such as a high radiation dose^(^^10^^,^^11^^)^. Although magnetic resonance imaging (MRI) can also be used for this purpose, it does not enable a conclusive diagnosis in the majority of cases^(^^10^^,^^12^^-^^16^^)^.

Chemical-shift MRI, also known as in-phase/out-of-phase imaging, has been found to be helpful in the evaluation of neoplastic lesions^(^^17^^-^^19^^)^. Signal intensity is derived from the sum of signals from lipid and water spins on in-phase images and from the difference between those signals on out-of-phase images^(^^18^^,^^19^^)^. Because the majority of tumors tend to replace the fatty and hematopoietic marrow components completely, these lesions show a persistence of signal intensity on out-of-phase images compared with in-phase images^(^^19^^,^^20^^)^. Therefore, decreased signal intensity on out-of-phase images compared with in-phase images indicates the presence of fat and water in bone marrow, rendering a neoplasm less likely^(^^18^^)^.

Some authors have described the use of relative ratios to evaluate chemical-shift MRI findings^(^^18^^)^. The relative ratio is calculated by dividing the out-of-phase signal intensity by the in-phase signal intensity. For the detection of neoplasms, a relative ratio cut-off value of 0.81 has shown a sensitivity of approximately 90%, whereas a relative ratio cut-off value of 1.2 has shown a specificity of approximately 90%^(^^17^^-^^19^^)^. The purpose of this study was to determine the usefulness of chemical-shift MRI in the diagnosis of osteoid osteoma by evaluating its ability to detect the nidus and surrounding inflammatory tissue.

## MATERIALS AND METHODS

### Study group

This retrospective study was approved by our institutional review board. The study group included 17 patients (11 males and 6 females), with a mean age of 18 years (range, 9-37 years), who had histologically confirmed osteoid osteoma, having been treated between January 2010 and February 2012. The mean duration of pain was 9 months (range, 4-12 months). Only one patient reported pain that was exacerbated at night and relieved by nonsteroidal anti-inflammatory drugs. Tumors were detected in the hip joint area in 11 patients (in the femoral head, in 7, and in the femoral diaphysis, in 4); in the tibial diaphysis, in 4; in the patellae, in 1; and in the humeral head, in 1.

### Imaging techniques

For nidus identification, all patients underwent CT, standard MRI sequences, and chemical-shift MRI, as part of our department’s standard protocol. The CT examinations were performed in a a 64-channel multislice scanner (Brilliance; Philips Medical Systems, Cleveland, OH, USA) with a field of view, matrix size, and slice thickness dependent on the specific site under study. The MRI scans-including T1-weighted spin-echo sequences, with a repetition time/echo time (TR/TE) of 443/15 ms; T2-weighted spin-echo sequences, with a TR/TE of 4390/109 ms; and fast multiplanar inversion recovery sequences, with a TR/TE of 4780/24 ms-were acquired in a 1.5 T scanner (Magnetom Avanto; Siemens, Erlangen, Germany). Fat-suppressed T1-weighted spin-echo sequences were also acquired before and after gadolinium administration (dynamic contrast-enhanced imaging). The field of view, matrix size, slice thickness, and choice of coils depended on the specific site under study. Fast multiplanar spoiled gradient-echo sequences, comprising in-phase images (TR/TE, 185/4.6 ms; flip angle, 90°) and out-of-phase images (TR/TE, 185/2.4 ms; flip angle, 70°), were also obtained for each patient.

### Chemical-shift MRI analyses

Signal intensities on in-phase and out-of-phase images were recorded using three circular regions of interest (ROIs) in each patient: the nidus of the tumor, identified as the nodular area inside the lesion, with low signal intensity on in-phase images and high signal intensity on out-of-phase images; bone marrow surrounding the lesion, with abnormal signal intensity; and normal-appearing bone marrow adjacent to the lesion. The ROIs were placed in identical locations on in-phase and out-of-phase images, and values were recorded three times each by two of the authors (FC and CC), with 15 and 5 years of experience, respectively, in musculoskeletal imaging. The mean value of the three measurements was considered to be the final value for each region studied. To assess intra-observer agreement, both radiologists performed a second reading of signal intensity values after a 6-week interval.

Relative signal intensity ratios were calculated for all ROIs. Relative ratios > 1 were considered indicative of neoplastic lesions, as described previously^(^^14^^)^. Relative ratios < 1 were considered indicative of the presence of lipid and water protons within the lesion and therefore of non-neoplastic status.

### Statistical analyses

The Mann-Whitney U test was used in order to standardize the sample. For each imaging method, inter- and intra-observer agreement were assessed using intraclass correlation coefficients (ICCs) according to the Fleiss method^(^^21^^)^. An ICC > 0.65 was considered indicative of substantial agreement, and values from only one reader were used in subsequent analyses.

Means, ranges, and standard deviations of relative signal intensity ratios for the three ROIs were calculated for each reader. The relative ratios of the nidus and abnormal-intensity surrounding bone marrow were compared with those of normal-appearing bone marrow using Student’s t-tests and Pearson’s correlation coefficient. Values of *p* < 0.05 were considered statistically significant.

## RESULTS

The means, ranges, and standard deviations for each ROI studied are presented in Table 1. No significant difference in relative signal intensity ratios due to patient gender or age was observed according the Mann-Whitney U test (*p* = 0.073). The relative ratios of the three ROIs showed substantial inter-observer agreement (nidus, ICC = 0.65; abnormal-intensity bone marrow, ICC = 1; normal-appearing bone marrow, ICC = 0.6) and intra-observer agreement (nidus, ICC = 0.65; abnormal-intensity bone marrow, ICC = 1; normal-appearing bone marrow, ICC = 0.65).

**Table 1:** Mean relative signal intensity ratios of normal-appearing bone marrow, abnormal-intensity bone marrow surrounding the lesion, and the nidus, on in-phase and out-of-phase images.

	Relative ratio	
ROI	Mean ± SD	Range
Normal bone marrow	0.93 ± 0.07	0.8-1.0
Abnormal bone marrow	0.35 ± 0.19	0.11-0.66
Nidus of the tumor	1.2 ± 0.1	0.9-1.4

SD, standard deviation.

**Figure 1:**
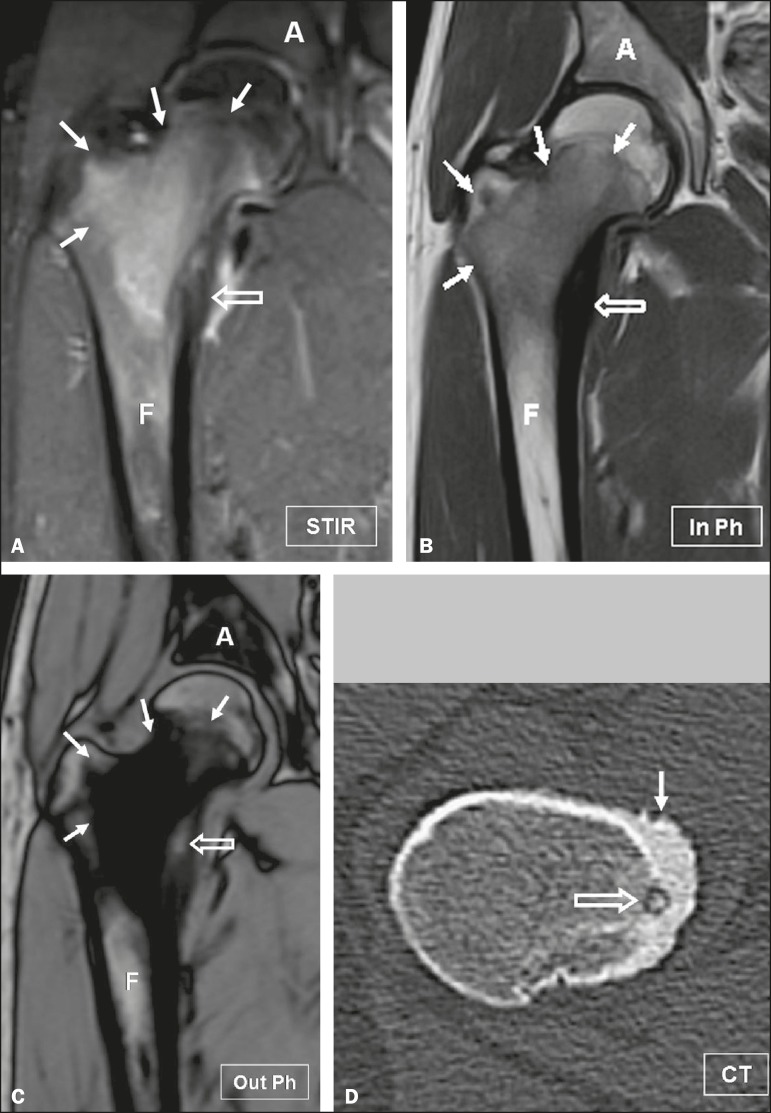
Osteoid osteoma in an 18-year-old male patient presenting with pain in the right hip. **A:** Coronal short inversion-time inversion recovery MRI sequence showing the high signal intensity of the lesion (arrows) and cortical thickening (open arrow). Coronal in-phase and out-of-phase spoiled gradient-echo MRI sequences (**B** and **C**, respectively) showing high signal intensity of a portion of the lesion (open arrow) on out-of-phase images, predicting the tumor nidus. Note the low signal intensity of the surrounding bone marrow (arrows), corresponding to inflammatory tissue. **D:** Reformatted axial computed tomographic image showing cortical thickening (arrow) and the nidus (open arrow), confirming the diagnosis of osteoid osteoma. A, acetabulum; F, femur.

**Figure 2:**
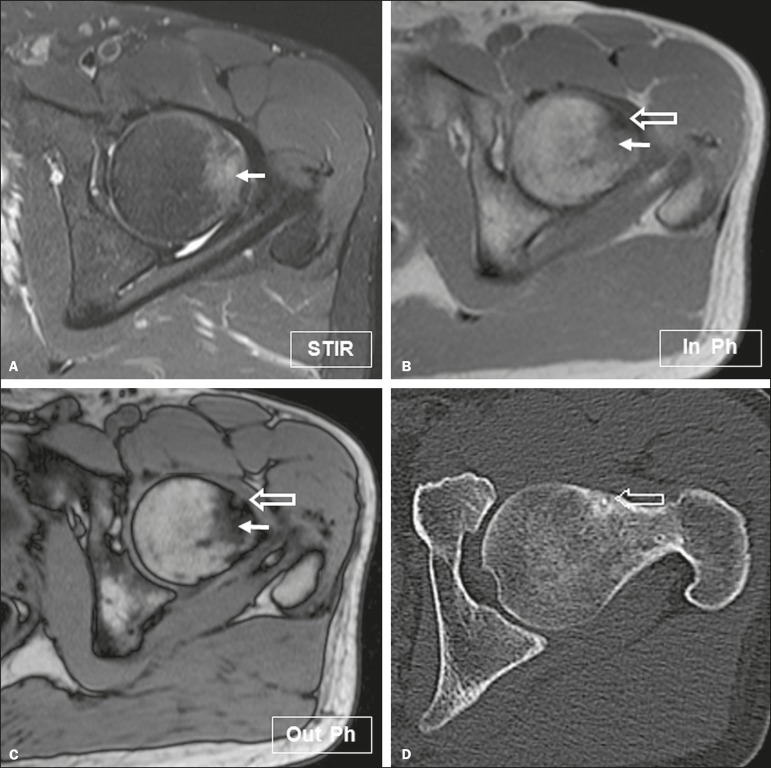
Osteoid osteoma in a 16-year-old male patient presenting with pain in the left hip. **A:** Axial short inversion-time inversion recovery MRI showing the high signal intensity of the lesion (arrow). Axial in-phase and out-of-phase spoiled gradient-echo MRI sequences (**B** and **C**, respectively) showing high signal intensity of the central portion of the lesion (open arrow) on out-of-phase image, predicting the tumor nidus. Note the low signal intensity of the surrounding bone marrow (arrows), corresponding to inflammatory tissue. **D:** Reformatted axial computed tomographic image showing the nidus (open arrow), confirming the diagnosis of osteoid osteoma.

Areas of variable signal intensity were observed on fast multiplanar inversion recovery sequences (Figures 1A and 2A). Gadolinium-enhanced T1-weighted images with fat saturation showed variable enhancement of the lesions and adjacent bone marrow. In all cases, nodular persistence of the signal intensity of the lesion was observed on out-of-phase images (Figures 1C and 2C) compared with in-phase images (Figures 1B and 2B). The mean relative signal intensity ratio in the nidus ROI was 1.2 (range, 0.9-1.4), predicting a neoplastic lesion. The relative ratios of the nidus were significantly higher than were those of normal-appearing bone marrow (*p* < 0.05).

Substantially reduced signal intensity of the abnormal-surrounding tissue was also observed on all out-of-phase images, and relative signal-intensity ratios of these regions were significantly higher than were those of normal-appearing bone marrow (*p* < 0.05). The mean relative signal intensity ratio for the surrounding tissue was 0.35 (range, 0.11-0.66), predicting a non-neoplastic lesion, probably of inflammatory origin.

## DISCUSSION

The radiology literature of Brazil has recently focused on the role of MRI in the diagnosis of musculoskeletal diseases^(^^22^^-^^30^^)^. The assessment of osteoid osteoma with MRI could be helpful. However, as confirmed in our study, this tumor exhibits variable signal intensity and contrast enhancement on standard MRI sequences. Chemical-shift MRI, first described by Wismer et al. in 1985, allows rapid interpretation of images by visual assessment and adds at most 4-5 min to the total imaging time^(^^31^^)^. The acquisition of in-phase and out-of-phase images allows the detection of fat in lesions and might thus be predictive of whether abnormal signal intensity in bone marrow is caused by a neoplastic or non-neoplastic lesion^(^^18^^)^. Fat replacement due to the neoplastic process results in similar signal intensities on in-phase and out-of-phase images, yielding relative ratios exceeding 0.81 and 1.2, respectively, as described previously^(^^20^^-^^33^^)^.

In our study, the relative ratios for the osteoid osteoma nidus were > 1 in all patients, allowing confirmation of the neoplastic origin of the lesions. In addition, substantially reduced signal intensities of surrounding tissue on out-of-phase images were demonstrated in all patients (mean relative ratio, 0.35), confirming the non-neoplastic origin of abnormal bone marrow signal intensity, which probably represented inflammatory tissue typically surrounding the tumor nidus, which replaces the normal hematopoietic marrow.

Although CT remains the technique of choice for nidus identification, we believe that chemical-shift MRI is an important sequence that aids this effort and contributes to the diagnosis of osteoid osteoma, because it is a radiation-free imaging modality. Dynamic contrast-enhanced perfusion MRI has also been used for nidus identification. Most osteoid osteomas show arterial phase enhancement and rapid partial washout as a result of hypervascularity of the nidus. Chemical-shift MRI does not requires gadolinium administration and can be used in patients with contraindications to contrast use. However, it should be borne in mind that many benign marrow lesions present a signal loss of < 20% on chemical-shift MRI, overlapping that of malignancy. In such cases, bone biopsy is necessary on order to make an accurate diagnosis^(^^34^^,^^35^^)^. At our facility, chemical-shift MRI is part of the protocol for the evaluation of osteoid osteoma, facilitating its diagnosis, especially when gadolinium cannot be administered.

As already described, the MRI features of osteoid osteoma can be misleading because bone marrow and soft-tissue changes associated with the tumor are sometimes be extensive. These features often lead to a diagnosis of osteomyelitis, stress fracture, or a more aggressive bone tumor^(^^9^^)^. Chemical-shift MRI has shown potential for application in the differential diagnosis between these lesions, because it allows the detection of the nidus.

We acknowledge that the small number of patients included in this analysis constitutes a limitation of our study. Another important limitation is the lack of any subjects presenting with diagnoses other than osteoid osteoma. Therefore, further studies are needed in order to confirm our preliminary results.

## CONCLUSION

Chemical-shift MRI, a widely available technique that allows rapid interpretation of images by visual assessment, is useful for the diagnosis and evaluation of osteoid osteoma.
